# Associations of cognitive appraisal and patient activation on disability and mental health outcomes: a prospective cohort study of patients undergoing spine surgery

**DOI:** 10.1186/s12891-024-07709-2

**Published:** 2024-07-29

**Authors:** Richard L. Skolasky, Joel A. Finkelstein, Carolyn E. Schwartz

**Affiliations:** 1 Departments of Orthopaedic Surgery and Physical Medicine & Rehabilitation, The Johns Hopkins University School of Medicine, 601 N. Caroline Street, Suite 5244, Baltimore, MD 21287 USA; 2 Department of Surgery, University of Toronto, Toronto, Canada; 3 Division of Orthopedic Surgery, Sunnybrook Health Sciences Centre, Toronto, ON Canada; 4 Division of Spine Surgery, Sunnybrook Health Sciences Centre, Toronto, ON Canada; 5 DeltaQuest Foundation Inc, Concord, MA USA; 6 Departments of Medicine and Orthopaedic Surgery, Tufts University Medical School, Boston, MA USA

**Keywords:** Quality of Life, Patient reported outcome measures, Spine, Orthopedic procedures, Response shift, Appraisal, Patient activation

## Abstract

**Background:**

With the increased use of patient-reported outcomes measures (PROMs) to assess spine surgery outcomes, it is important to understand how patients interpret their health changes over time. The measurement of cognitive-appraisal processes enables the quantification of how individuals think about quality of life (QOL). This study examined how appraisal processes were associated with patients’ views of their role in managing their health—patient activation.

**Methods:**

This longitudinal cohort study from August 2019 to January 2022 included 222 adults undergoing spine surgery for cervical (*n* = 107) and/or lumbar (*n* = 148) pathology at an academic medical center. PROMs assessed disability (Neck Disability Index for cervical or Oswestry Disability Index for lumbar) and mental health (PROMIS-29 v2.0), cognitive-appraisal processes (QOLAP_v2_-SF), and patient activation (Patient Activation Measure). ANOVA models were used to examine the relationships between QOL and cognitive appraisal processes before and after surgery, overall and stratified by patient-activation stage. Effect sizes facilitated interpretation.

**Results:**

There were significant improvements in pain-related disability and mental health following surgery. Cognitive appraisal processes explained substantial amounts of variance, particularly with changes in mental health (45% before surgery, 75% at three months, and 63%, at 12-months after surgery). With respect to physical disability, less disability was associated with a lesser focus on negative aspects of QOL. Appraisal explained the most variance before surgery for high-activation patients. At 12-months post-surgery, however, appraisal explained the most variance for the low-activation patients. Appraisal explained similar amounts of variance in mental health at baseline and three-months post-surgery for all activation groups, but substantially more variance in the low-activation group at 12-months post-surgery. There were differences in the direction of appraisal-outcome associations by activation group in selected appraisal items/domains.

**Conclusions:**

Cognitive-appraisal processes demonstrate a significant relationship with QOL among spine surgery patients. These processes explain substantial variance in pain-related disability and mental health, especially among those high in activation before surgery and those low in activation at 12-months post-surgery. Our findings suggest that patients’ ways of thinking about their health may be effective targets of motivational coaching, to help them become more engaged over the recovery trajectory.

**Supplementary Information:**

The online version contains supplementary material available at 10.1186/s12891-024-07709-2.

## Introduction

Patient-reported outcome measures (PROMs) in spine surgery research provide an important perspective in evaluating treatment outcomes, particularly in the reporting of pain and pain-related disability. Like many aspects of health-related quality of life (QOL), pain and pain-related disability are subjective and thus best described by the patient [1]. As with any evaluative, subjective outcome, they are influenced by how an individual patient thinks about their health. *Cognitive-appraisal processes* refer to how people characterize QOL within four domains that address individual differences in how people think about their QOL: Frame of Reference (QOL definition, Goal delineation), Sampling of Experience, Standards of Comparison, and Combinatory Algorithm (i.e., patterns of emphasis) [2–5]. These cognitive-appraisal processes are important in understanding treatment impact and burden in a broad range of medically ill populations [5–9].

Cognitive-appraisal processes also provide an understanding of patient adaptation over time. When experiencing a change in health status, one may change their internal standards, values, and/or conceptualization of symptoms or other aspects of QOL (i.e., recalibration, reprioritization, or reconceptualization) [10–13]. These underlying changes are termed *response-shift* effects [10, 14], and challenge a basic assumption of PROMs: that the meaning of an item to a respondent does not change over time (measurement invariance) [15]. Interpreting change over time is thus complicated by this fundamental human process of adaptation. Research on cognitive-appraisal processes over time in the context of spine surgery research has documented that patients’ evaluation of treatment benefits, and even the magnitude of clinically important differences, are influenced by their appraisals and changes in appraisals in spine outcomes [16]. Indeed, appraisal processes were the most important predictors of spine and hip outcomes in studies using machine learning prediction models [17, 18]. These findings support the use of personalized interventions to encourage more adaptive appraisals following orthopaedic surgery [19, 20].

Although this emerging evidence base supports the importance of cognitive-appraisal processes in spine outcomes, it is less well understood how such appraisal processes relate to patient activation. Patient activation refers to how a patient views their role in managing their health condition [21–25]. For example, an individual with high patient activation understands the nature of their health condition, feels confident asking their healthcare provider questions (even if not asked), and can make behavior changes to improve their health. Thus, patient activation has been conceptualized as an individual’s propensity to engage in adaptive health behaviors that may lead to improved health outcomes [21]. As a theory of health behavior, patient activation moves the focus of attention to the individual and assesses the influence of psychological factors and personal competencies, such as condition-specific knowledge, on health behavior [21]. An activated patient is armed with the skills, knowledge, and motivation to be an effective member of the healthcare team [26]. It has been suggested that movement from one stage of patient activation to the next (detailed below) requires a specific set of psychological strengths, such as self-efficacy, and personal competency skills, such as problem-solving skills. Further, it has been hypothesized that as an individual moves through the stages of patient activation, his or her propensity to engage in adaptive health behavior increases [27, 28]. High patient activation is associated with better engagement in physical therapy and home exercise programs [23]. Further, interventions to improve patient activation lead to better health behavior and outcomes following spine surgery [25, 29, 30].

The current paper seeks to understand how cognitive-appraisal processes intersect with patient activation, specifically with regard to physical- and mental-health recovery after spine surgery. This work builds on previous work examining appraisal and spine surgery outcomes [17, 19] by explicitly considering the role of patient activation. We hypothesized that the association of appraisal with physical- and mental-health, improvement after surgery would be stronger among more activated patients.

## Materials and methods

### Study population

**Fig. 1:**
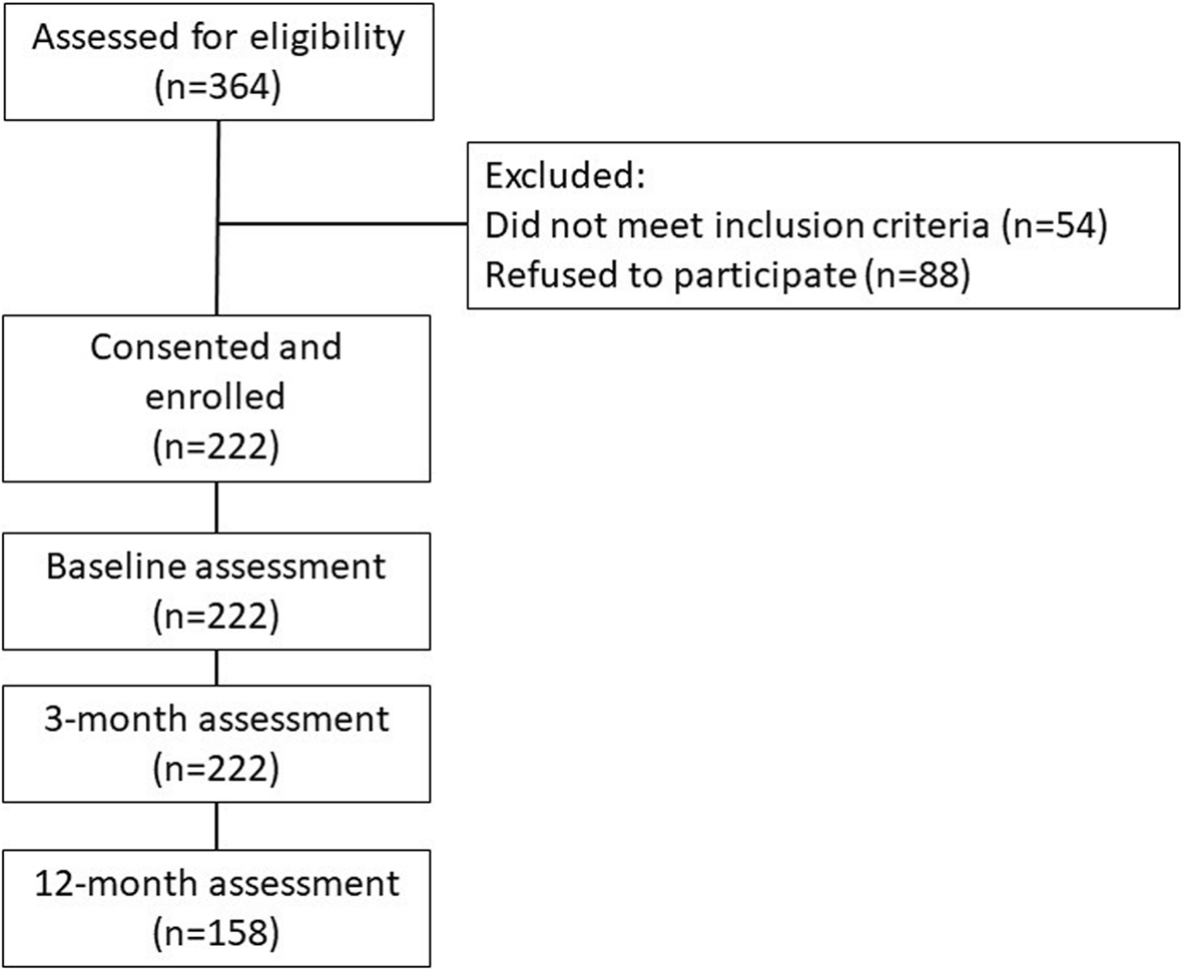
Study enrollment flow chart and sample retention

Patients presenting for evaluation and possible primary surgical procedures for cervical and/or lumbar degenerative spine pathology at our academic medical center between December 2020 and November 2021 were eligible for this prospective cohort study. Eligible patients were English-speaking adults (age ≥ 18 years). We excluded patients with revision procedures, trauma, infection, or tumor as these patients typically present with more severe disease, require more invasive surgical procedures, and experience different trajectories in recovery (see flow chart Fig. 1).

The Johns Hopkins School of Medicine Institutional Review Board approval was received, and patient informed consent was provided before participation. Participants were responsible for their treatment costs and did not receive compensation for their participation in this study. Participants were free to seek additional care (e.g., physical therapy) for their spine pathology following surgery.

### Baseline measures

Self-reported social determinants of health were age, gender, educational attainment, and household income. Educational attainment was stratified as < bachelor’s degree, bachelor’s degree, and post-graduate education. Household income was stratified as < $30,000, Between $30,000 and $80,000, and > $80,000 per year.

The presence of comorbid health conditions was assessed using a self-report version of the Charlson Comorbidity Index (CCI) [31]. Patients reported whether a doctor or healthcare provider had ever told them that they had: myocardial infarction, congestive heart failure, peripheral vascular disease, cerebrovascular disease (except hemiplegia), dementia, chronic pulmonary disease, arthritis or other connective tissue diseases, ulcer disease, mild liver disease, diabetes (without complications), diabetes with end organ damage, hemiplegia, moderate or severe renal disease, solid tumor (non-metastatic), leukemia, lymphoma or multiple myeloma, moderate or severe liver disease, metastatic solid tumor, or AIDS. We used the Elixhauser scoring algorithm to estimate 10-year survival [32].

Patient activation has been conceptualized as having an impact on six dimensions: (1) self-management of symptoms, (2) engagement in activities to maintain function, (3) involvement in healthcare decisions, (4) collaboration with healthcare providers, (5) informed choices of provider based on quality, and (6) navigation of the healthcare system [21]. This can be thought of as an individual’s propensity to engage in positive health behavior. Promoting patient activation is a core component of patient-centered care [33]. In our study, patient activation was assessed using the 13-item Patient Activation Measure (PAM) [34]. For each of the 13 items, patients are provided five response options, ranging from Strongly Agree to Strongly Disagree. Based on the answers, patients are assigned a numerical score ranging from 0 to 100, which can then be used to stratify patients into one of four hierarchical stages of activation: Stage 1 (believes taking an active role is important), Stage 2 (confidence and knowledge to take action), Stage 3 (taking action), and Stage 4 (staying the course under stress) [34]. The PAM is a reliable and valid assessment of patient activation in a cohort of individuals about to undergo surgery for low back pain and other health populations [24, 35, 36].

### Longitudinal measures

Prior to surgery, patients completed the Oswestry Disability Index (ODI) [37–39] or Neck Disability Index (NDI) [40, 41] and the Patient Reported Outcomes Measurement Information System (PROMIS)-29_v2_.0 profile [42, 43].

Disability was assessed using the Oswestry Disability Index (ODI) for patients with lumbar spine pathology, the Neck Disability Index (NDI) for patients with cervical spine pathology, and the maximum of the two measures for patients with both lumbar and cervical spine pathology. The ODI is a 10-item measure of low back pain–related disability that assesses the current effects of a patient’s low back pain on various aspects of daily living. ODI scores range from 0 to 100, with higher scores indicating greater disability [37–39]. The NDI is constructed similarly but focuses on neck-related disability [40, 41].

The PROMIS-29_v2_.0 profile assesses Pain Intensity using a single 11-item numeric rating scale and assesses seven health domains (Pain Interference, Physical Function, Fatigue, Anxiety, Depression, Sleep Disturbance, and Ability to Participate in Social Roles and Activities) using 4 items using a 5-point Likert scale (e.g., “never”, “rarely”, “sometimes”, “often”, “always”, or “not at all”) [42]. The response timeframe was “in the past 7 days”. Scores for each health domain are reported on a T-score metric (mean, 50; SD, 10 points) centered on the mean of a sample that matched the 2000 US census with respect to age, sex, race, and education [43]. Responses from the PROMIS-29_v2_.0 profile were used to calculate the PROMIS Mental Health Summary Scores (MHS), with higher scores indicating better mental health [44].

Before surgery (before the day of surgery), patients completed the Quality of Life Appraisal Profile, version 2 Short-Form (QOLAP_v2_-SF). The QOLAPv2-SF is a 28-item measure of cognitive-appraisal processes involved in answering QOL measures across four domains: 1) frame of reference; 2) experience sampling; 3) standards of comparison; and 4) combinatory algorithm (hereafter referred to as “patterns of emphasis”) [45]. The QOLAP_v2_-SF uses closed-ended rating-scale items ranging from “not at all like me” (1) to “very much like me” (5) or “not applicable/decline” (-99) [46]. Frame of reference (6 items) refers to the personal goals that matter most to the individual patients, such as “I want to reduce the amount of day-to-day help I need from others”. Sampling of experience (4 items) refers to recall and sampling of salient experiences, such as “recall recent episodes or flare-ups.” Patterns of emphasis (9 items) assesses the use of a subjective algorithm to prioritize and combine appraisals into an QOL rating, such as “the negative things that happened were more important than the positives.” Standard of comparisons (9 items) provides insight on the standards of comparison that a patient uses when thinking about their QOL, such as “a time in the past before you had your health condition” [2, 46]. The QOLAPv2-SF has been developed and validated in studies with over 6000 medically ill patient samples, including spine surgery, multiple sclerosis, heterogeneous chronic illnesses, heterogeneous cancer, bladder cancer, and human immunodeficiency virus [18, 45].

### Statistical analysis

We summarized the socio-demographic, clinical, and surgical characteristics of the patient population using descriptive statistics. All descriptive values are reported as Mean ± Standard Deviation. We examined the association of patient activation with disability and mental health outcomes using Pearson correlation coefficients as well as mixed-effects linear regression, adjusting for age, gender, and comorbidity risk score.

To understand how cognitive-appraisal processes may influence physical and mental health, Analysis of Variance (ANOVA) models were used. The ANOVA models examined the explained variance, direction, and magnitude of association (i.e., beta coefficients) with pain-related disability and mental health outcomes (i.e., the ODI or NDI or MHS as dependent variables in separate models) by individual items within domains of the QOLAP_v2_SF (independent variables). We examined these models at three time points: before surgery and three- and 12-months after spine surgery. Due to item distributions, appraisal items were collapsed into three categories: “Not at all/somewhat not like me” vs. “A little like me” vs. “Somewhat/Very much like.”

We then examined the influence of patient activation on the relationship between cognitive appraisal and pain-related disability and mental health. We stratified the sample into baseline stages of patient activation and estimated the variance explained, direction, and magnitude of association in physical and mental health outcomes by individual items on the QOLAP_v2_-SF before, and at three- and 12-months after spine surgery. Due to the small number of participants endorsing Stages 1 (*n* = 11) or 2 (*n* = 25) of patient activation, these groups were collapsed into a single category.

Effect sizes were used to facilitate interpretation, using Cohen’s cut-offs for explained variance (eta-squared, η^2^) and beta coefficients [47]. Tables were conditionally formatted to highlight the small, medium, and large effect size of the magnitude of η^2^ estimates and the magnitude of the estimated beta coefficients. More saturation reflects a larger effect size. Within domains of the QOLAP_v2_-SF, we computed the average η^2^ estimates to demonstrate the effect size for that domain based on prior work demonstrating low intercorrelation between individual items of the QOLAP_v2_-SF [48]. The magnitude of the beta coefficients was only interpreted if the η^2^ effect size was at least small.

Statistical analyses were performed using StataBE version 17 (StataCorp LLC, College Station, TX).

## Results

### Patient characteristics

We assessed cognitive appraisal in 222 patients presenting for evaluation of treatment options for cervical (*n* = 107) and lumbar (*n* = 148) spine problems [NOTE: Some patients could have both cervical and lumbar spine problems]. The mean age was 60 ± 18 years. Most patients identified as non-Hispanic White (84%) and female sex (52%) and reported high household income (55% reporting ≥ $80,000 per year). Most patients (66%) received a fusion procedure (36% with accompanying decompression). Overall, a majority (54%) of patients were in the highest stage of patient activation (Table 1).

**Table 1:** Sociodemographic and Clinical Characteristics

Characteristic	Overall (*n* = 222)	Spine pathology^a^	
		Cervical (*n* = 107)	Lumbar (*n* = 148)
Age, years (mean, sd)	60 ± 18	59 ± 16	61 ± 18
Female sex (no., %)	115 (52)	60 (56)	80 (54)
Race (no., %)			
non-Hispanic White	177 (84)	82 (82)	120 (85)
non-Hispanic Black	19 (9)	10 (10)	13 (9)
Hispanic	15 (7)	8 (8)	8 (3)
Education (no., %)			
Less than bachelor's degree	80 (36)	39 (36)	58 (39)
Bachelor's degree	71 (32)	32 (30)	49 (33)
Greater than bachelor's degree	71 (32)	36 (34)	41 (28)
Lives alone(no., %)	61 (27)	33 (31)	41 (28)
Household Income^b^ (no., %)			
< $30,000	21 (12)	8 (9)	17 (14)
$30-$80,000	60 (33)	32 (36)	41 (34)
≥$80,000	100 (55)	50 (55)	62 (52)
Current smoker, yes (no., %)	18 (8)	11 (7)	10 (9)
Charlson (no., %)	0.60 (0.34)	0.59 (0.35)	0.60 (0.32)
Disability^d^ (no., %)	43.4 ± 18.0	33.6 ± 18.6	47.5 ± 16.0
None	5 (2)	0 (0)	0 (0)
Mild	40 (18)	8 (23)	18 (12)
Moderate	88 (41)	11 (31)	60 (41)
Severe	67 (31)	14 (40)	55 (38)
Complete	17 (8)	2 (6)	12 (8)
PROMIS-29 v2.0 (mean ± sd)			
Mental Health	42.8 ± 7.7	42.2 ± 7.5	43.6 ± 7.9
Diagnosis^c^ (no., %)			
Disc herniation	13 (6)	12 (8)	2 (2)
Stenosis	83 (37)	70 (47)	20 (19)
Spondylosis	41 (18)	10 (7)	33 (31)
Spondylolisthesis	25 (11)	24 (16)	3 (3)
Radiculopathy	82 (37)	48 (32)	40 (38)
Myelopathy	52 (23)	48 (45)	6 (4)
Primary Procedure (no., %)			
Decompression alone	39 (18)	26 (18)	14 (13)
Fusion alone	67 (30)	34 (23)	43 (40)
Decompression and fusion	81 (36)	62 (42)	34 (32)
Patient Activation (no., %)			
Stage 1 or 2	36 (16)	16 (15)	24 (16)
Stage 3	67 (30)	35 (33)	47 (32)
Stage 4	119 (54)	56 (52)	77 (52)

*SD* Standard deviation

^a^An individual could endorse more than spine pathology

^b^Household income available on 181 patients

^c^An individual could have more than one diagnosis

^d^Disability was assessed using the Oswestry Disability Index for patients with lumbar spine pathology, the Neck Disability Index for patient with cervical spine pathology, and the maximum of the two measures for patients with both lumbar and cervical spine pathology

There were 222 (100%) participants assessed at 3-months and 152 (68%) participants assessed at 12-months.

### Change in patient-reported outcomes

**Fig. 2:**
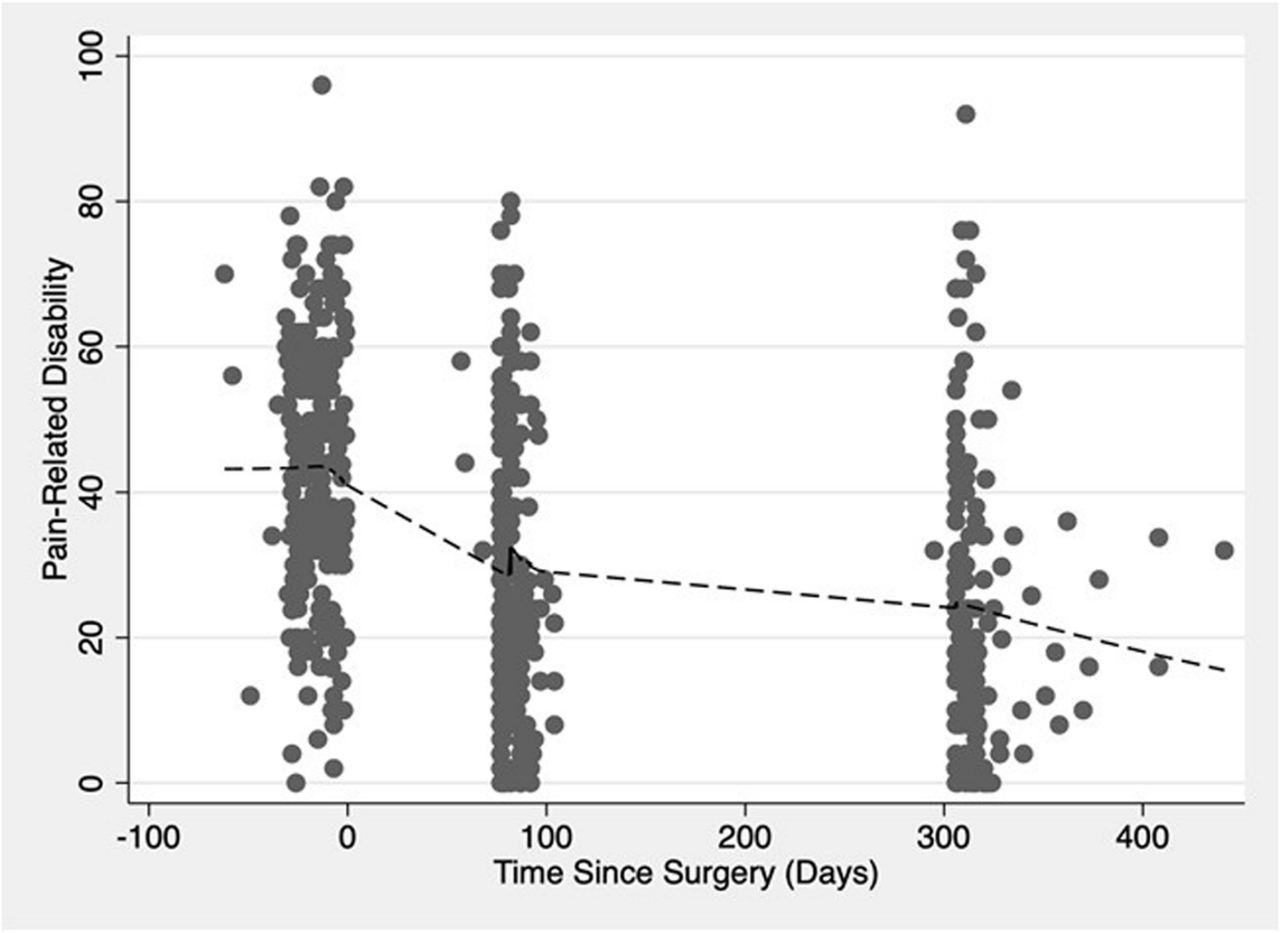
Scatter plot of Oswestry/Neck Disability Index with locally weighted smoothing lines to demonstrate improvement by Time Since Surgery (Days)

**Fig. 3:**
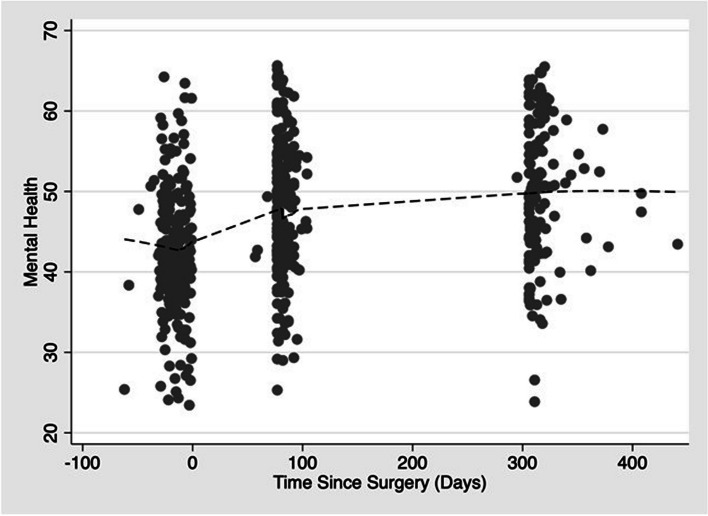
Scatter plot of PROMIS-29, version 2.0 Mental Health Summary Score with locally weighted smoothing lines to demonstrate improvement by Time Since Surgery (Days)

There were significant improvements in pain-related disability following surgery using mixed-effects linear regression (Table 2). After adjusting for age, gender, and CCI using the Elixhauser score, patients reported a 17.2-point (95% confidence interval (CI), 14.5, 19.9) reduction in the ODI and 9.3-point (95% CI, 6.8, 11.9) reduction in NDI at three-months following surgery. Patients reported improvement in mental health with the patients experiencing, on average, a 5.2-point (95% CI, 4.3, 6.0) improvement in MHS at three months compared to before surgery. Similar improvements in pain-related disability and mental health were observed at 12-months following surgery compared to before surgery. These improvements seen following surgery are consistent with published thresholds for minimally important differences in pain-related disability [49, 50] and mental health [51, 52]. Locally Weighted Scatterplot Smoothing (lowess) lines show trends over time on these two outcome measures (Figs. 2 and 3).

**Table 2:** Change in Patient-Reported Outcome Measures

PRO Measure	Before Surgery	After Surgery (Months)		*p*-value^*^	
		3	12Ɨ	Before-to-3 months	Before-to-12 months
Disability^***^					
ODI	47.5 ± 16.0	30.2 ± 19.3	25.8 ± 20.1	< .001	< .001
NDI	33.6 ± 18.6	24.3 ± 17.2	19.6 ± 18.3	< .001	< .001
PROMIS-29 v2.0					
MHS	42.9 ± 7.6	48.08 ± 8.2	49.9 ± 8.7	< .001	< .001

*MHS* Mental Health Summary Score, *NDI* Neck Disability Index, *ODI* Oswestry Disability Index, *PRO* Patient-Reported Outcome, *PROMIS*-*29 v2.0* Patient Reported Outcome Measurement Information System 29 item Health Profile (version 2.0)

^*^P-value based on mixed effects linear regression, adjusting for age, gender, and Elixhauser comorbidity index

^Ɨ^There were 100/148 (68%) patients with lumbar spine pathology and 67/107 (63%) patients with cervical spine pathology with 12-month follow-up.

### Association of patient activation with disability and mental health outcomes

At all time points, higher patient activation was associated with lower disability and better mental health using linear regression. The associations at pre-surgery had small effect sizes, whereas at three- and 12-months post-surgery, the associations had medium effect sizes (see Supplementary Table 2).

### Pain-related disability

Table 3. shows the explained variance (η^2^) and beta coefficients from ANOVA models predicting pain-related disability using the ODI or NDI before and at three- and 12-months following surgery. By summing the average η^2^ for each appraisal domain, it is notable that appraisal processes explained a total of 20% of the variance in disability before surgery (Frame of reference, 0.05 + Sampling of experience, 0.06 + Combinatory algorithms, 0.07 + Standards of comparison, 0.02), and 44% (0.17 + 0.12 + 0.12 + 0.03) and 40% (0.11 + 0.16 + 0.07 + 0.06) at three- and 12-months post-surgery, respectively.

**Table 3.**
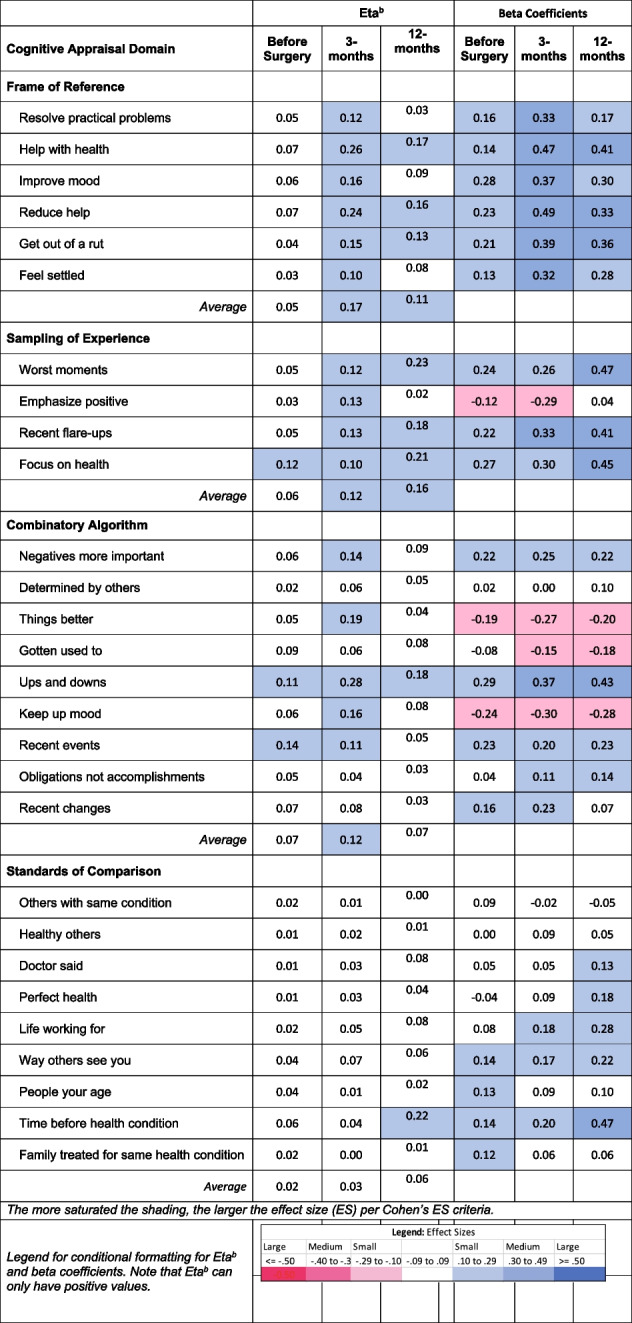
Effect size estimates (η^2^ and beta coefficients) for Cognitive Appraisal Processes on Pain-Related Disability Before and At Three- and 12-months after Spine Surgery

^1^ODI, Oswestry Disability Index, was used for lumbar spine patients and NDI, Neck Disability Index, was used for cervical spine patients

^2^Frame of reference occurs when a person recalls experiences that they deem relevant to their response

^3^Sampling of experience occurs when a person samples specific experiences within their frame of reference to formulate a response

^4^Combinatory algorithm occurs when a person summarizes their evaluation of relevant experiences and formulates a response

^5^Standards of comparison provides insight on the comparators that a patient uses when thinking about their health

Disability is the maximum of ODI or NDI to account for those with both lumbar and cervical pathology (taking the most affected pathology)

Disability has been recoded to reflect that higher scores reflect less disability

#### Frame of reference

 Frame of Reference explained on average small, large, and then medium amounts of variance on pain-related disability before surgery and at three- and 12-months, respectively (average η^2^: 0.05, 0.17, and 0.11, respectively). All of the beta coefficients were positive, suggesting that engaging more in frame-of-reference appraisals was associated with worse disability, which was particularly salient at three-months post-surgery. The items most implicated at three-months post-surgery (when there was a large effect size in explained variance) related to focusing on getting Help with Health, Improving Mood, Reducing Help Needed, and Getting Out of a Rut.

#### Sampling of Experience

 Sampling of Experience explained medium, medium, and large amounts of variance in pain-related disability over time (average η^2^: 0.06, 0.12, and 0.16, before surgery and at three- and 12-months, respectively). The beta coefficients were positive for all but one of the items, suggesting that engaging more in experience-sampling appraisals focused on negative aspects of health was associated with worse disability at all time points but particularly at 12-months post-surgery. In contrast, greater emphasis on the positive at baseline and three-months post-surgery was associated with less disability.

#### Combinatory Algorithm

Combinatory Algorithm (patterns of emphasis) explained medium amounts of variance at all time points (average η^2^: 0.07, 0.12, and 0.07 before surgery and at three-, and 12-months, respectively). The beta coefficients were negative for focusing on things getting better, habituating, and keeping up mood, suggesting that engaging more in such appraisals was associated with less disability. In contrast, focusing more on the negatives, recent events, obligations, and recent changes were associated with more disability. The items most implicated in worse outcomes at three- and 12-months (explained large amounts of the variance in disability) were focusing on Negatives More Important and Ups and Downs, and not focusing on Things Getting Better and Keeping Up Mood.

#### Standards of Comparison

 Standards of Comparison explained negligible amounts of variance before surgery and at three months post-surgery, but a medium effect size at 12-months post-surgery (average η^2^: 0.02, 0.03, and 0.06, before surgery and at three-, and 12-months, respectively). The beta coefficients at 12-months post-surgery were most often small and positive and revealed that focusing more on the Time Before the Health Condition was associated with worse pain-related disability. This same item was most implicated in worse outcomes at 12-months (explaining large amounts of the variance in disability).

### Influence of patient activation on association of cognitive appraisal with disability

**Fig. 4:**
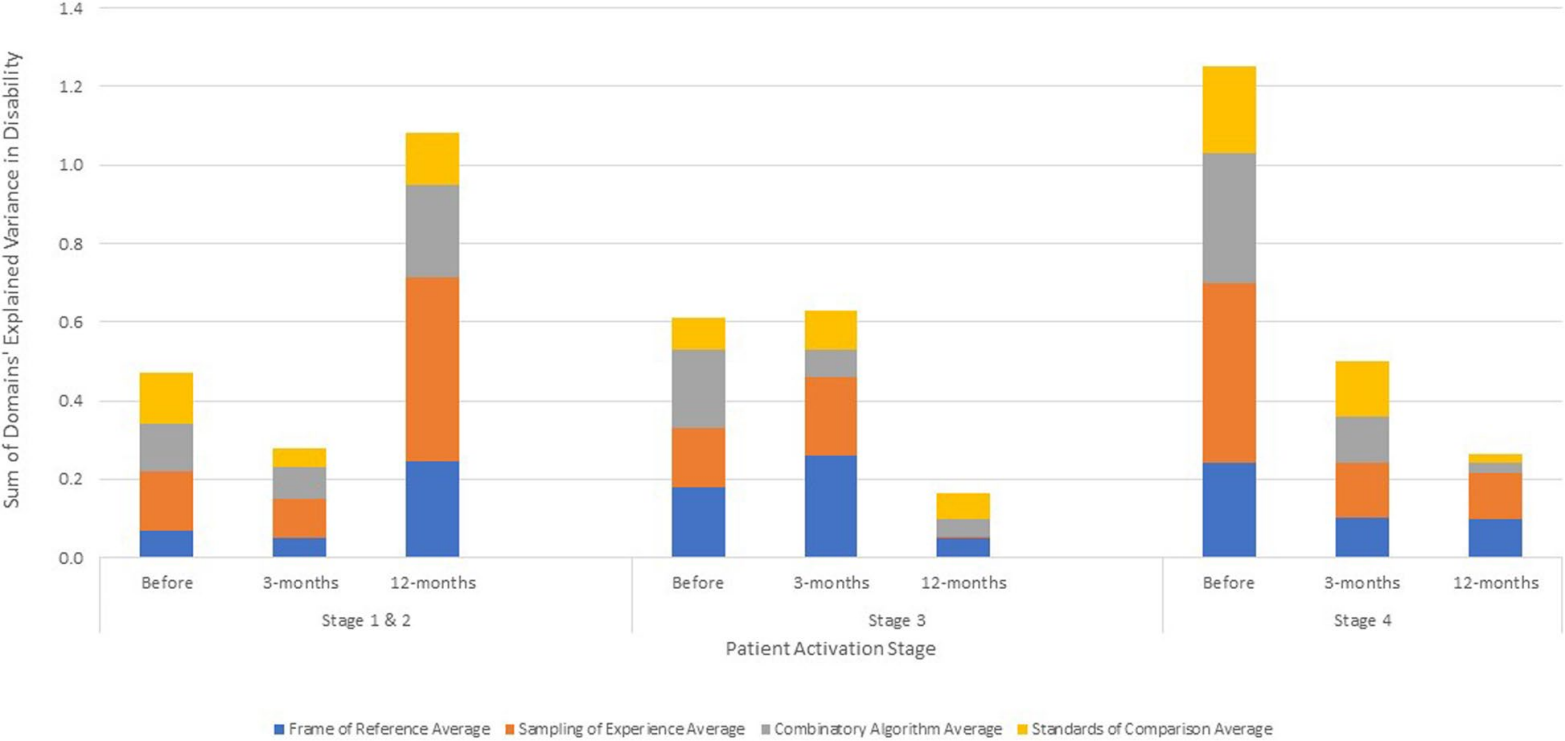
Appraisal-explained Variance in Disability Over Time by Patient Activation Stage

Table 4. shows the explained variance (η^2^) and beta coefficients from ANOVA models stratified by patient-activation stage, predicting pain-related disability using the ODI or NDI before and at three- and 12-months following surgery. Before surgery, appraisal explained substantially more variance in disability among patients at the highest stage of activation (i.e., Stage 4 (staying the course under stress)) than at lower levels (i.e., Stages 1 (believes taking an active role is important), 2 (confidence and knowledge to take action) or 3 (taking action)) (Fig. 4). At three- and 12-months following surgery, appraisal explained much less than at baseline but comparable amounts of variance in disability among these most activated patients. The direction of association of appraisal items within domains was similar across levels of patient activation for frame of reference, experience sampling, and patterns of emphasis, but differed for standards of comparison, particularly for the low-activation patients (Stages 1 and 2) compared to the others.

**Table 4.**
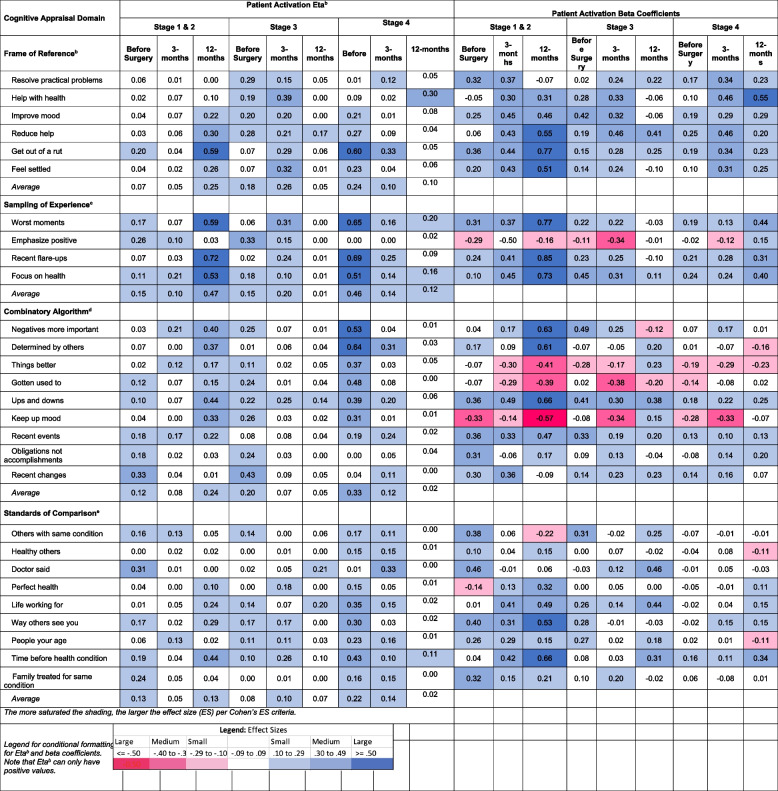
Explained Variance (η^2^) and Standardized Beta Coefficients for Cognitive Appraisal Processes on Pain-Related Disability^1^ Before and At Three- and 12-months after Spine Surgery, Stratified by Stage of Patient Activation

^1^ODI, Oswestry Disability Index, was used for lumbar spine patients and NDI, Neck Disability Index, was used for cervical spine patients

^2^Frame of reference occurs when a person recalls experiences that they deem relevant to their response

^3^Sampling of experience occurs when a person samples specific experiences within their frame of reference to formulate a response

^4^Combinatory algorithm occurs when a person summarizes their evaluation of relevant experiences and formulates a response

^5^Standards of comparison provides insight on the comparators that a patient uses when thinking about their health

^6^Patient Activation assessed using the 13-item Patient Activation Measure with Stages 1 and 2 combined due to sample size

Disability is the maximum of ODI or NDI to account for those with both lumbar and cervical pathology (taking the most affected pathology)

Disability has been recoded to reflect that higher scores reflect less disability

### Mental health

Table 5. shows the explained variance η^2^ and beta coefficients from ANOVA models predicting mental health before and at three- and 12-months following surgery. Appraisal processes explained a total of 45% of the variance in mental health before surgery, 75% at three-months post-surgery, and 63% at 12-months post-surgery.

**Table 5.**
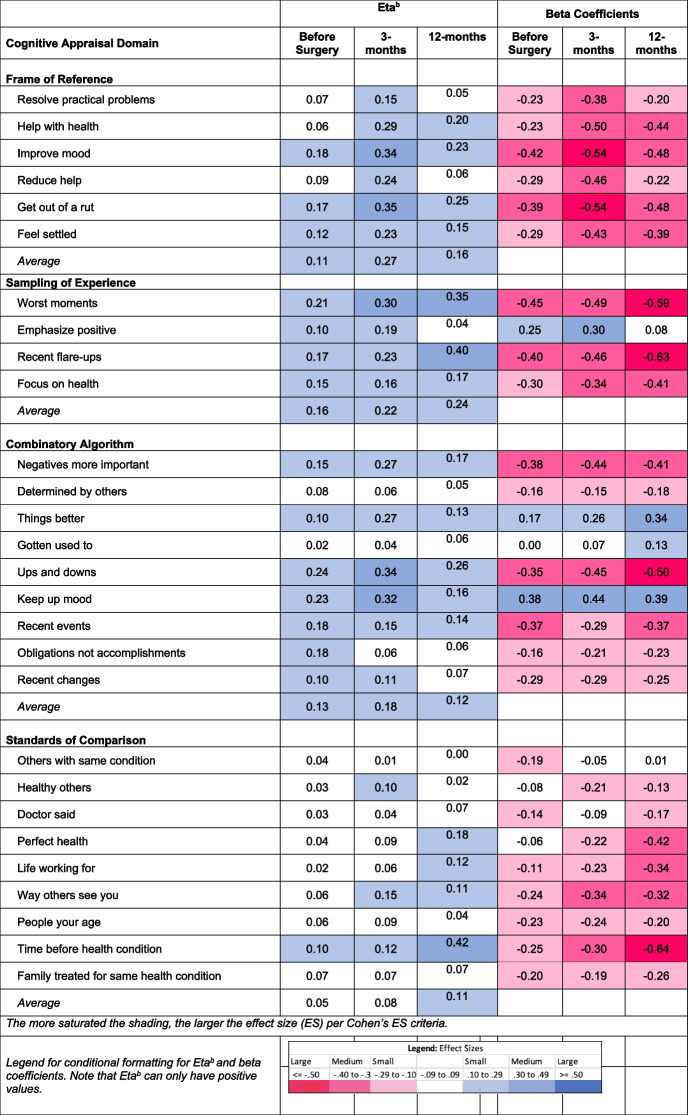
Effect size estimates (η^2^) for Cognitive Appraisal Processes on Patient-Reported Mental Health Problems Before and At Three- and 12-months after Spine Surgery

^1^MHS, Mental Health Score from the PROMIS-29 v2.0 health profile

^2^Frame of reference occurs when a person recalls experiences that they deem relevant to their response

^3^Sampling of experience occurs when a person samples specific experiences within their frame of reference to formulate a response

^4^Combinatory algorithm occurs when a person summarizes their evaluation of relevant experiences and formulates a response

^5^Standards of comparison provides insight on the comparators that a patient uses when thinking about their health

#### Frame of Reference

 Frame of Reference explained medium, large, and then large amounts of variance in mental health before surgery and at three- and 12-months, respectively (average η^2^: 0.11, 0.27, and 0.16, respectively). All of the beta coefficients were large and negative, suggesting that engaging less in frame-of-reference appraisals was associated with worse mental health. The items most implicated at three-months post-surgery were Help with Health, Improve Mood, and Get Out of a Rut.

#### Sampling of Experience

Sampling of Experience explained large amounts of variance in mental health at all times (average η^2^: 0.16, 0.22, and 024, at baseline, three-, and 12-months, respectively). The beta coefficients were large and negative for all but one of the items, suggesting that engaging less in experience-sampling appraisals focused on negative aspects of health was associated with worse mental health at all time points, particularly throughout recovery. In contrast, focusing more on emphasizing the positive, particularly at three-months post-surgery, was associated with better mental health.

#### Combinatory Algorithm

Combinatory Algorithm (patterns of emphasis) explained medium, large, and medium amounts of variance before surgery and at three-, and 12-months post-surgery (average η^2^: 0.13, 0.18, and 0.12, respectively). Among those appraisal processes that explained large amounts of variance, the beta coefficients were large and negative for focusing on Negatives More Important, Ups and Downs, and Recent Events, suggesting that engaging less in such appraisals was associated with better mental health. In contrast, focusing more on Things [Getting] Better at three-months post-surgery, and on Keeping up Mood at all time points were associated with better mental health.

#### Standards of Comparison

Standards of Comparison explained small, medium, and medium amounts of variance before surgery and at three- and 12-months post-surgery, respectively (average η^2^: 0.05, 0.08, and 0.11, before surgery and at three-, and 12-months, respectively). The beta coefficients at 12-months post-surgery were large and generally negative and revealed that focusing less on the Way Others See You at three-months post-surgery, and less on Perfect Health and the Time Before the Health Condition were associated with better mental health.

### Influence of patient activation on association of cognitive appraisal with mental health

**Fig. 5:**
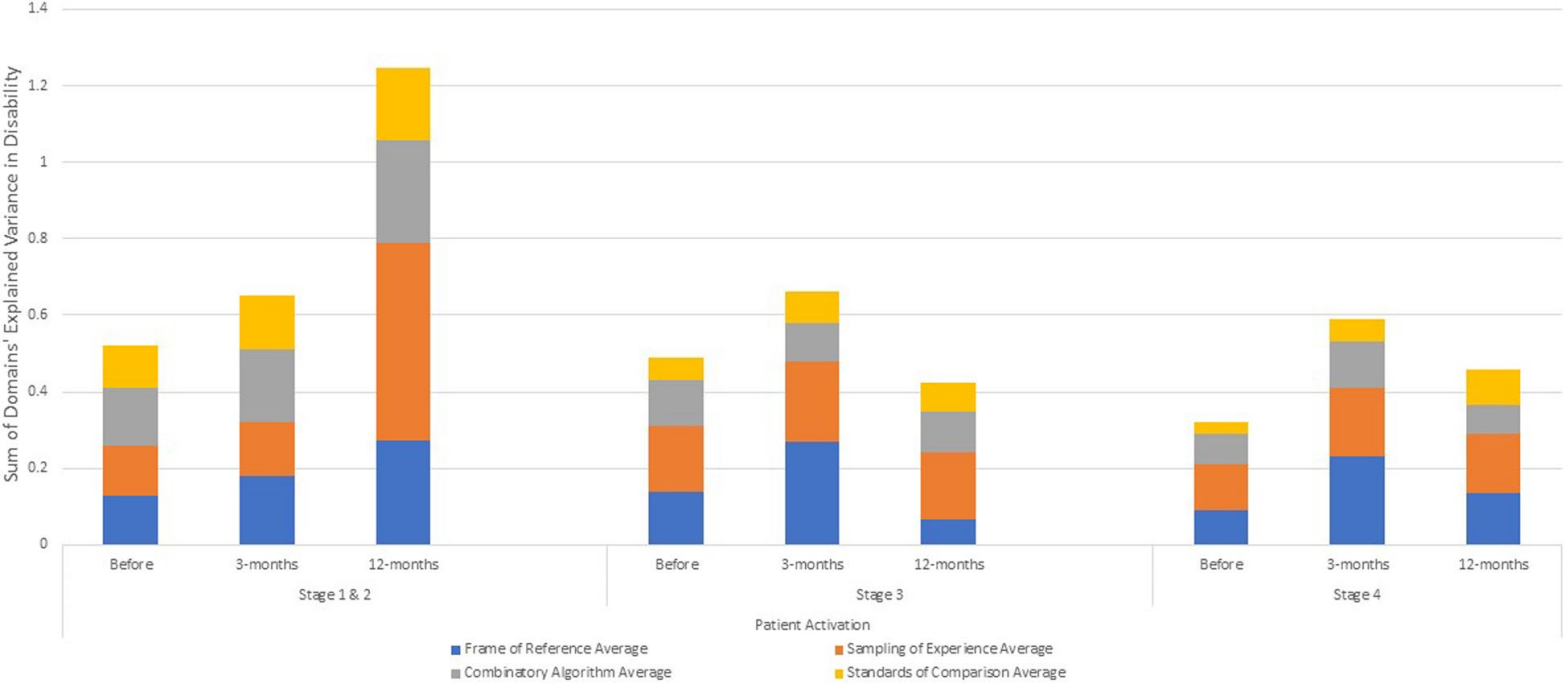
Appraisal-explained Variance in Mental health Over Time

Table 6. shows the explained variance (η^2^) and beta coefficients from ANOVA models stratified by patient-activation stage, predicting mental health before and at three- and 12-months following surgery. In contrast to disability, explained variance in mental health was similar by stage of activation over time with one exception: at 12-months post-surgery, appraisal explained substantially more variance among patients at the lowest stages of activation (i.e., Stages 1 and 2) as compared to the other two stage-groups (Fig. 5). The direction of association of appraisal items within domains was similar across levels of patient activation for frame of reference, experience sampling, and patterns of emphasis, but differed for standards of comparison for specific items within activation groups.

**Table 6.**
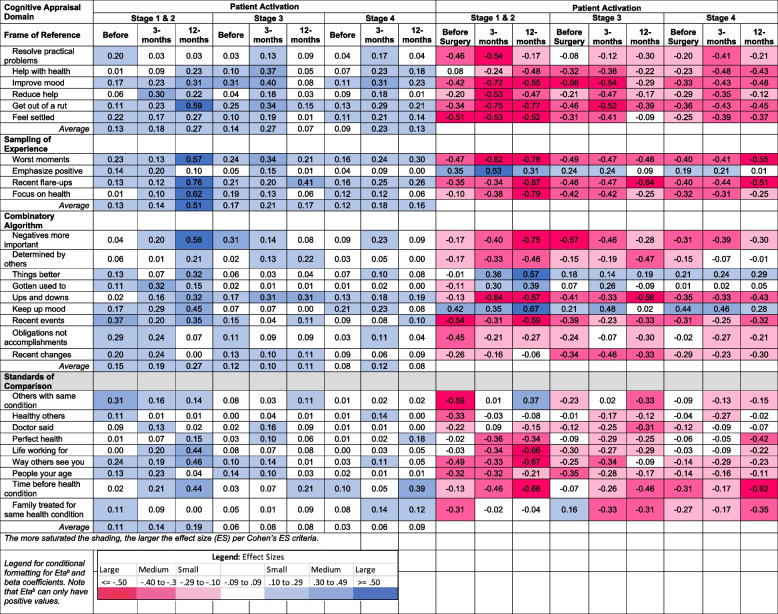
Explained Variance (η^2^) and Standardized Beta Coefficients for Cognitive Appraisal Processes on Patient-Reported Mental Health Problems Before and At Three- and 12-months after Spine Surgery, Stratified by Stage of Patient Activation

^1^MHS, Mental Health Score from the PROMIS-29 v2.0 health profile

^2^Frame of reference occurs when a person recalls experiences that they deem relevant to their response

^3^Sampling of experience occurs when a person samples specific experiences within their frame of reference to formulate a response

^4^Combinatory algorithm occurs when a person summarizes their evaluation of relevant experiences and formulates a response

^5^Standards of comparison provides insight on the comparators that a patient uses when thinking about their health

^6^Patient Activation assessed using the 13-item Patient Activation Measure with Stages 1 and 2 combined due to sample size

Disability is the maximum of ODI or NDI to account for those with both lumbar and cervical pathology (taking the most affected pathology)

Disability has been recoded to reflect that higher scores reflect less disability

The direction of association of appraisal items within domains was similar across levels of patient activation for Frame of Reference, Sampling of Experience, and Combinatory Algorithm, but differed for Standards of Comparison for specific items within activation groups. Among the low-activation group, comparing themselves to what the doctor said was associated with worse mental health before surgery and better mental health at 12-months post-surgery (ns at three months). Among the medium (stage 3) activation group, comparing themselves to other family members with the same health condition was associated with better mental health before surgery, but worse mental health at three- and 12-months post-surgery. Thus, the variation in the impact of the same standard-of-comparison appraisal process was found for mental health, with differences revealed for each patient-activation subgroup.

## Discussion

This study demonstrated that cognitive-appraisal processes had a strong relationship with QOL among spine surgery patients. These processes explained less variance in pain-related disability than mental health both before and following spine surgery. This difference is consistent with other studies of appraisal in spine surgery patients [16, 17, 19]. Increased endorsement of each item within each appraisal domain was generally associated with higher levels of disability and lower levels of mental health across patient-activation strata. For example, increased focus on reducing help from others was associated with worse disability and worse mental health. In general, appraisal processes related to getting help, remaining independent, and acknowledging the struggles of recovery were associated with lesser disability and better mental health.

Subgroup analyses that examined the intersection of patient activation and appraisal pointed to several important take-home messages. For low-activation patients, cognitive-appraisal processes were more important in explaining disability and mental health scores later in the recovery trajectory (i.e., at 12 months post-surgery). In contrast, for high-activation patients, cognitive-appraisal processes were more important in explaining disability before surgery but were similarly important across all time points with regard to mental health.

### Clinical implications

Overall, these findings indicate that patients’ ways of thinking about their health may be effective targets of motivational coaching. Developing interventions to target patient’s way of thinking may help them become more engaged over the recovery trajectory. Patient activation has a clinically important association with surgical outcomes, an association that increases after surgery. Addressing specific cognitive-appraisal processes has the potential to influence outcomes following spinal surgery in both the disability and mental health domains.

Low-activation patients are those patients who, based on their responses to the Patient Activation Measure, do not believe that the patient role is important or do not have the confidence or knowledge necessary to take action with respect to their own healthcare [21]. This is reflected by less of a connection between appraisal and both disability and mental-health outcomes. These patients seem to be relying more on external forces to fix the problem (i.e., the surgeon), rather than their own sense of agency to partner towards a solution. Coaching low-activation patients could focus on increasing awareness of the different appraisal processes. Armed with this awareness, surgeons could partner with other clinicians to develop interventions that incorporate strategies to increase patients’ level of activation that may improve motivation and adherence to post-surgical rehabilitation.

### Limitations

The present work is part of a larger investigation of factors that influence recovery following spine surgery. As such, it benefits from the prospective collection of patient demographics, clinical, and surgical characteristics, and patient-reported outcome measures collected at clinically relevant time points before and after surgery. The current analysis was focused on the associations among cognitive-appraisal processes and health outcomes over time, examining in particular up to 12-months post-surgery. We found that these associations differed over time, so it is possible that longer follow-up may also reveal different associations over time. Future work might examine these associations at two years following surgery. Additionally, our patient sample is overwhelming non-Hispanic white, older, and of higher socio-economic status. These characteristics; however, are consistent with other large observational studies of patients seeking care for spine pathology and undergoing spine surgery [53–55]. In addition, most of our patients (54%) endorse the highest level of patient activation. The U.S. healthcare system is complex. For patients to present for spine surgery, they have been able to navigate the complex system and have the resources and support to undergo an invasive surgical procedure that often requires a lengthy recovery. The conclusions drawn from this sample may not be generalizable to other patient populations.

## Conclusions

Cognitive-appraisal processes explain substantial variance in pain-related disability and mental health among spine surgery patients, especially among those high in activation before surgery and those low in activation at 12-months post-surgery. Increased endorsement of each item within each appraisal domain was generally associated with higher levels of disability and lower levels of mental health across patient-activation strata. Our findings suggest that patients’ ways of thinking about their health may be effective targets of motivational coaching. Such motivational coaching could help them become more involved in their healthcare and become more resilient and adherent to physical therapy and home exercise during early recovery from spine surgery.

### Supplementary Information

Supplementary Material 1.

## Data Availability

The datasets generated and/or analyzed during the current study are not publicly available due to their confidentiality but are available from the corresponding author upon reasonable request.
