# Supplementary material for: Associations of cognitive appraisal and patient activation on disability and mental health outcomes: a prospective cohort study of patients undergoing spine surgery
Source: BMC Musculoskelet Disord. 2024 Jul 29;25:595. doi: 10.1186/s12891-024-07709-2 (PMC11285205; doi:10.1186/s12891-024-07709-2)
Supplement: Supplementary file 1 — Supplementary Material 1. [file 12891_2024_7709_MOESM1_ESM.docx]

| **Supplemental Table 1**  **.** Descriptive Statistics of Quality of Life (QOL) Appraisal, version 2 (QOLAP-v2) items of 222 Patients Presenting for Surgical Treatment of a Cervical and/or Lumbar Spine Condition | | | |
| --- | --- | --- | --- |
| **Item Number** | **Item** | **Mean** | **Standard Deviation** |
| Frame of Reference | | | |
| 1 | Resolve practical problems | 2.84 | 1.65 |
| 2 | Help with health | 4.01 | 1.25 |
| 3 | Improve mood | 3.44 | 1.55 |
| 4 | Reduce help | 3.9 | 1.43 |
| 5 | Get out of a rut | 3.54 | 1.5 |
| 6 | Feel settled | 3.15 | 1.59 |
| Sampling of Experience | | | |
| 7 | Worst moments | 2.61 | 1.01 |
| 8 | Emphasize positive | 3.89 | 0.93 |
| 9 | Recent flare-ups | 2.75 | 1.08 |
| 10 | Focus on health | 3.84 | 0.96 |
| Combinatory Algorithm | | | |
| 11 | Negatives more important | 2.22 | 1.19 |
| 12 | Determined by others | 2.33 | 1.34 |
| 13 | Things better | 3.2 | 1.21 |
| 14 | Gotten used to | 3.08 | 1.19 |
| 15 | Ups and downs | 2.84 | 1.29 |
| 16 | Keep up mood | 3.96 | 1.12 |
| 17 | Recent events | 3.28 | 1.27 |
| 18 | Obligations not accomplishments | 3.63 | 1.1 |
| 19 | Recent changes | 3.72 | 1.14 |
| Standards of Comparison | | | |
| 20 | Others with same condition | 1.96 | 0.93 |
| 21 | Healthy others | 2.71 | 1.2 |
| 22 | Doctor said | 3.06 | 1.07 |
| 23 | Perfect health | 3.24 | 1.15 |
| 24 | Life working for | 3.59 | 0.97 |
| 25 | Way others see you | 2.96 | 1.11 |
| 26 | People your age | 3.07 | 1.06 |
| 27 | Time before health condition | 3.28 | 1.13 |
| 28 | Family treated for same health condition | 2.41 | 1.21 |

| **Supplementary Table 2.** Correlation and Regression Betas for Patient Activation and Outcomes | | | |
| --- | --- | --- | --- |
|  | Bivariate Pearson Correlation | Beta coefficient after adjusting for age, gender, comorbidity risk score | Effect Size Magnitude |
| **Pre-operatively** |  |  |  |
| Disability | -0.17 | -0.19 | small |
| Mental Health | 0.24 | 0.11 | small |
| **3 months Post-Op** |  |  |  |
| Disability | -0.37 | -0.36 | medium |
| Mental Health | 0.4 | 0.16 | small |
| **12 months Post-Op** |  |  |  |
| Disability | -0.33 | -0.33 | medium |
| Mental Health | 0.35 | 0.15 | small |
